# Impaction grafting in the femur in cementless modular revision total hip arthroplasty: a descriptive outcome analysis of 243 cases with the MRP-TITAN revision implant

**DOI:** 10.1186/1471-2474-14-19

**Published:** 2013-01-11

**Authors:** Matthias D Wimmer, Thomas M Randau, Moritz C Deml, Rudolf Ascherl, Ulrich Nöth, Raimund Forst, Nadine Gravius, Dieter Wirtz, Sascha Gravius

**Affiliations:** 1 Department of Orthopaedics and Trauma Surgery, University of Bonn, Bonn, Germany; 2 Department of Orthopaedics and Trauma Surgery, Zeisigwaldkliniken Bethanien, Chemnitz, Germany; 3 Department of Orthopaedic Surgery, König-Ludwig-Haus, University of Würzburg, Würzburg, Germany; 4 Waldkrankenhaus St. Marien, Department of Orthopedics, University of Erlangen, Erlangen, Germany

**Keywords:** Arthroplasty, Hip, Revision, Modular, Impaction bone grafting

## Abstract

**Background:**

We present a descriptive and retrospective analysis of revision total hip arthroplasties (THA) using the MRP-TITAN stem (Peter Brehm, Weisendorf, GER) with distal diaphyseal fixation and metaphyseal defect augmentation. Our hypothesis was that the metaphyseal defect augmentation (Impaction Bone Grafting) improves the stem survival.

**Methods:**

We retrospectively analyzed the aggregated and anonymized data of 243 femoral stem revisions. 68 patients with 70 implants (28.8%) received an allograft augmentation for metaphyseal defects; 165 patients with 173 implants (71.2%) did not, and served as controls. The mean follow-up was 4.4 ± 1.8 years (range, 2.1–9.6 years). There were no significant differences (p > 0.05) between the study and control group regarding age, body mass index (BMI), femoral defects (types I-III as described by Paprosky), and preoperative Harris Hip Score (HHS). Postoperative clinical function was evaluated using the HHS. Postoperative radiologic examination evaluated implant stability, axial implant migration, signs of implant loosening, periprosthetic radiolucencies, as well as bone regeneration and resorption.

**Results:**

There were comparable rates of intraoperative and postoperative complications in the study and control groups (p > 0.05). Clinical function, expressed as the increase in the postoperative HHS over the preoperative score, showed significantly greater improvement in the group with Impaction Bone Grafting (35.6 ± 14.3 vs. 30.8 ± 15.8; p ≤ 0.05). The study group showed better outcome especially for larger defects (types II C and III as described by Paprosky) and stem diameters ≥ 17 mm. The two groups did not show significant differences in the rate of aseptic loosening (1.4% vs. 2.9%) and the rate of revisions (8.6% vs. 11%). The Kaplan-Meier survival for the MRP-TITAN stem in both groups together was 93.8% after 8.8 years. [Study group 95.7% after 8.54 years ; control group 93.1% after 8.7 years]. Radiologic evaluation showed no significant change in axial implant migration (4.3% vs. 9.3%; p = 0.19) but a significant reduction in proximal stress shielding (5.7% vs. 17.9%; p < 0.05) in the study group. Periprosthetic radiolucencies were detected in 5.7% of the study group and in 9.8% of the control group (p = 0.30). Radiolucencies in the proximal zones 1 and 7 according to Gruen occurred significantly more often in the control group without allograft augmentation (p ≤ 0.05).

**Conclusion:**

We present the largest analysis of the impaction grafting technique in combination with cementless distal diaphyseal stem fixation published so far. Our data provides initial evidence of improved bone regeneration after graft augmentation of metaphyseal bone defects. The data suggests that proximal metaphyseal graft augmentation is beneficial for large metaphyseal bone defects (Paprosky types IIC and III) and stem diameters of 17 mm and above. Due to the limitations of a retrospective and descriptive study the level of evidence remains low and prospective trials should be conducted.

## Background

The long-term success of femoral revision arthroplasty depends on several factors. First, the joint’s physiologic biomechanics should be restored by reconstructing the anatomic center of rotation. Then, the implant fixation should provide initial stability and allow full weight bearing. A third and crucial factor is the biological reconstruction of femoral bone defects, to restore a functional implant bed capable of bearing physiologic loads [1], and to downgrade the femoral defect situation to facilitate a possible subsequent revision procedure [2].

The consensus in the current literature is that implant fixation simply by filling the femoral defects with bone cement leads to poor long-term results and should not be recommended [3]. The bone cement cannot provide an intrusive, interlocking bond in a smooth-walled osteolytic femoral canal [4]. Cemented fixation can be combined with Impaction Bone Grafting [5-8]. The femoral canal is filled with bone grafts to obtain a normally dimensioned implant bed, then the implant is cemented [9]. The success rate of this method varies in the literature [10-12]. Potential disadvantages include impaired union due to cement penetration into the graft [13] and the increased risk of postoperative femoral fractures at the level of osteolytic areas [14].

Cementless revision implants were initially developed as single component implants, but modular designs have become increasingly popular [15,16]. Modular implant systems provide greater variability in difficult anatomical situations compared to monoblock revision stems. The modular design can be adapted to the individual surgical setting, allowing nearly physiologic joint reconstruction [17].

Cementless revision stems are available with two different fixation concepts. Revision stems with a rough proximal surface and metaphyseal fixation promote proximal stress transfer, counteracting stress shielding and progressive bone loss. Even though metaphyseal stem fixation can be difficult to achieve in the presence of extensive proximal osteolysis. Results are often unsatisfactory in such cases [18,19]. On the other hand, stems that employ the initial distal diaphyseal press fit concept entail a risk of stress shielding in the proximal femur [15,20]. We present the surgical technique and the descriptive mid-term follow-up results of the modular MRP-TITAN revision stem (Peter Brehm, Chirurgie-Mechanik, Weisendorf, Germany) with initial distal diaphyseal fixation with or without supplementary metaphyseal defect augmentation.

## Methods

### Patients

The results of 265 femoral revisions performed in 255 patients were followed in a multicenter patient registry. Patients were treated in four centers focusing on primary and revision THA in Germany. All patients received the MRP-TITAN stem. Inclusion criteria were revision THA with bony defects of the proximal femur graded as Paprosky I- III. Exclusion criteria included all kinds of tumors or secondary neoplasia diseases, NYHA IV and ASA IV. A total of 8 surgeons performed the operations. All surgeons were experienced attendings and board certified orthopedic-surgeons doing more than 100 primary or revision THAs a year. 22 patients were lost to follow up, thus clinical and radiographic findings were evaluated in 233 patients with 243 implants with a follow-up period of at least 2 years. 68 out of these patients with 70 implants (28.8%) received an allograft augmentation of metaphyseal defects using the implant-specific Impaction Bone Grafting System (Peter Brehm, Chirurgie-Mechanik, Weisendorf, Germany). 165 patients with 173 implants (71.2%) did not receive metaphyseal defect augmentation and served as controls.

Our hypothesis was that Impaction Bone Grafting improves stem survival. The endpoint of our study was revision surgery of the stem. Patients were retrospectivly and descriptivly analysed using aggregated and anonymized data.

The decision whether the Impaction Bone Grafting technique was used or not was made intraoperativly by the surgeon based on clinical criteria such as defect size, defect location and containment. A relevant selection bias could be excluded because both groups, patients with and without IBG, did not vary significantly according to age, sex, weight, height, body mass index (BMI), intraoperative femoral defects as described by Paprosky et al. [21], implant stem diameter, and preoperative function as measured by the HHS (Table 1).

**Table 1:** Patient data in the study and control groups

	**Study group (n = 70)**	**Control group (n = 173)**	**p value**
**Patient data**			
Age [years]	69.2 ± 9.9	67.1 ± 9.8	0.14
Sex [m / f]	23 / 47	69 / 104	0.31
(% male)	32.9	39.9	
Weight [kg]	73.4 ± 11.4	76.7 ± 14	0.08
Height [m]	1.66 ± 0.09	1.68 ± 0.1	0.12
BMI [kg / m2]	26.2 ± 3.9	26.7 ± 4.4	0.42
HHS preop.	35.5 ± 14.3	39.4 ± 15.7	0.07
**Femoral defects (I – III Paprosky classification) [intraoperative findings]**			
Type I [n] (%)	4 (5.7)	12 (6.9)	0.73
Type II A [n] (%)	20 (28.6)	55 (31.8)	0.62
Type II B [n] (%)	15 (21.4)	33 (19.1)	0.68
Type II C [n] (%)	12 (17.1)	35 (20.2)	0.58
Typ III [n] (%)	19 (27.1)	38 (22)	0.39
**MRP-TITAN stem diameter**			
13 / 14 mm [n] (%)	10 (14.3)	22 (12.7)	0.74
15 / 16 mm [n] (%)	21 (30)	56 (32.4)	0.72
17 / 18 mm [n] (%)	24 (34.3)	59 (34.1)	0.98
19 / 20 mm [n] (%)	13 (18.6)	35 (20.2)	0.77
≥ 21 mm [n] (%)	2 (2.9)	1 (0.6)	0.15

### Clinical evaluation

The mean follow-up period was 4.4 ± 1.8 years (min.-max. 2.1–9.6 years). Clinical evaluation included the duration of surgery, duration of hospitalization, time of mobilization, time to full weight bearing, intraoperative and postoperative complications, improvement of the HHS, and the causes of revision surgery and implant failure.

### Radiographic evaluation

Analogue, standardized plain pelvis radiographs with a scale of 1:1.15 and a Lauenstein view of the symptomatic hip were routinely obtained preoperatively. The follow up radiographs were evaluated in a blinded fashion for radiologic signs of implant loosening according to the criteria published by Kavanagh and Fitzgerald [22]. Proximal femoral bone defects were evaluated according to the classification described by Paprosky et al. [21].

Evaluation of follow-up radiographs included assessment of signs of loosening or periprosthetic radiolucencies in zones 1–7 according to Gruen as well as secondary axial implant migration, varus or valgus tilt, and rotation. Implants showing axial migration of more than 5 mm, progressive signs of osteolysis or complete periprosthetic radiolucency were evaluated as unstable or loosened. Implant tilting was determined comparing the angle between the longitudinal axis of the femoral diaphysis and the implant stem. Changes of more than 5 degrees occurring over the observed course were regarded as significant. Stem rotation was evaluated by comparing the radiologic landmarks of the implant with the femur landmarks. Periprosthetic bone regeneration, bone resorption, progressive radiolucencies, and visible osteolysis were evaluated according to the criteria described by Engh et al. [23]. Periarticular ossification was evaluated using the criteria described by Brooker et al. [24]. All measurements were done manually by a blinded investigator, not knowing whether IBG was used or not.

### Implant

The modular MRP-TITAN revision stem is a system based on modular taper connections designed for cementless implantation with initial distal diaphyseal fixation. The implant components are made of a titanium alloy (TiAl6Nb7) with a rough corundum-blasted surface with a pore size of 40–60 μm to facilitate osseous integration.

The modular design essentially consists of three components:

– The distally tapered femoral stem with longitudinal parabolic ribs for fixation with rotational stability. The stem is available as a straight-stem model in 140 mm and 200 mm length, and curved-stem version to fit the physiologic anterior bow of the femur in 200 mm length. 260 mm and 320 mm curved stems with two distal transverse drill holes are also available, providing the possibility to use distal locking bolts for additional stability. Diameters are available in 1 mm increments between 13 and 22 mm.

– An optional extension sleeve, adding 30 mm to the stem length.

– Three different neck models (sizes 50, 60, and 70 mm) with a standard taper (Euro taper 12/14). The neck components are available with different neck-stem angles of 130° (37 mm offset) and 123° (47 mm offset).

The core of the system is a taper connection using optimized materials. This connection permits individual adjustment of the angle of femoral anteversion and axial coupling of the individual components. The continuously adjustable taper connections are locked intraoperatively with extension bolts at a torque of 25 Nm, using an implant-specific torque wrench. The modular design allows intraoperative adaption of total implant length from 190 mm to 420 mm with freely adjustable rotation.

**Figure 1:**
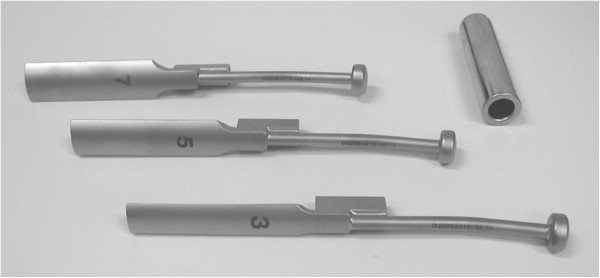
**Impaction grafting system (IGS).** Special impactors in sizes 3, 5 and 7 mm and extension sleeve (Peter Brehm GmbH, Weisendorf, Germany) to be used for the proximal metaphyseal allograft augmentation, using the press fit impaction technique.

A special impactor (diameter 3, 5 or 7 mm) is available as an additional instrument for proximal metaphyseal allograft augmentation, using the press fit impaction technique (impaction grafting system, IGS, Figure 1).

The implant is designed to facilitate diaphyseal implant fixation and covers the indications as follows:

– Stem loosening with deficient osteolytic bone stock (type I-III femoral defects as described by Paprosky).

– Implant fractures, fractures distal to the implant, and periprosthetic fractures.

– Bone loss following tumor surgery.

Due to the principle of the implant, extensive metaphyseal and diaphyseal defects with cortical thinning and a wide diaphyseal medullary canal, which would render diaphyseal press fit fixation difficult (type IV femoral defects as described by Paprosky), must be considered a contraindication.

### Surgical technique

The surgical procedure was modified from the previously described technique according to Mumme et al. [25]. First, the stem of the primary implant was removed. If a cemented stem was in place, the proximal cement was removed with an osteotome. The distal cement mantle was then removed with reamers of different diameters under continuous fluoroscopic control. Occasionally the stem could not be removed in this manner or cement residues remained. In these cases, a rectangular cortical window was opened in the femoral shaft over the residual cement and the implant was removed through the femur. The bone removed to create the cortical window was replaced and fixed afterwards. The femoral canal was then reamed with flexible reamers of increasing sizes. Reaming continued until the reamer was in full contact with the bone on all sides within a circular canal over a distance of 7–10 cm in the distal diaphyseal fixation zone. Next, a trial stem of the appropriate length and diameter (diameter 2–3 mm larger than the last reamer used) was inserted using the impactor/extractor with the central guide wire. The trial stem was inserted according to the physiologic anterior bow of the femur and far enough distally to achieve a diaphyseal press fit over a length of 7–10 cm. After determination of the definite size of the distal stem component, the appropriate implant neck length was measured, using the greater trochanter as a landmark. The selected neck trial was then attached over the guide wire and the desired rotation was selected. A trial head was attached and a trial reduction performed. Once the proper implant placement was determined in fluoroscopy and by clinical examination, the trial was removed and the definitive stem of the selected length and diameter was implanted.

**Figure 2:**
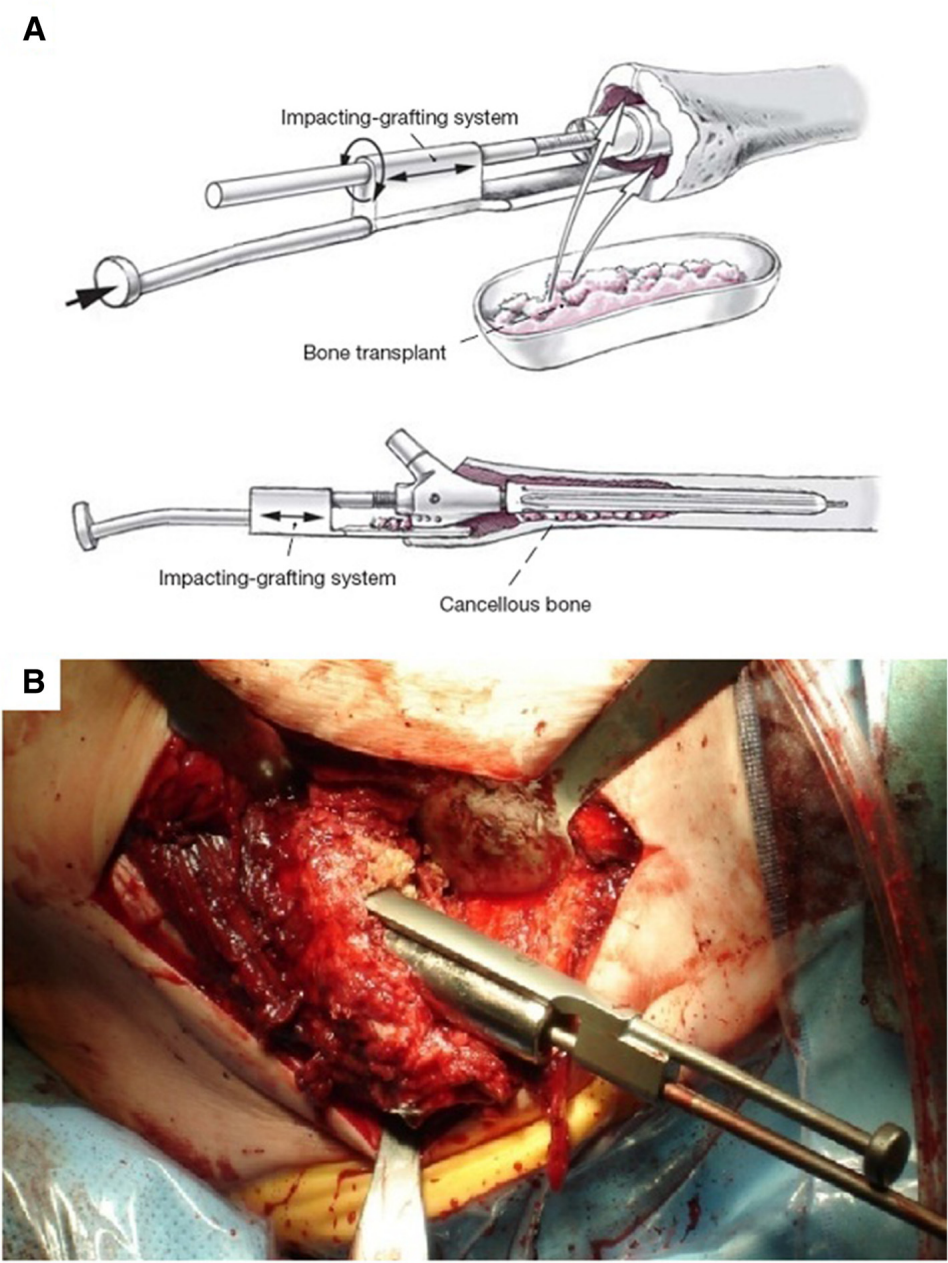
**Surgical technique.** Filling metaphyseal bone defects by impaction bone grafting, using the press fit impaction technique. Special impactors fit over the central guide rod and laterally “bypass” the trial implant, permitting meticulous compression of the allograft chips introduced into the metaphysis. (**A**.) Illustration from [25]; (**B**) intraoperative photograph.

The study group received proximal defect augmentation with impaction grafting. Commercially available allograft bone chips with an average size of 5–10 mm^3^ were produced with Luer forceps or with a bone grinder. The technique avoided the use of a heat-producing saw. The bone grafts were introduced into the metaphysis in layers. Each layer was meticulously compressed using the impaction grafting system (IGS) with impactors in sizes of 3, 5, and 7 mm. Care was taken to obtain a uniform mixture of various particle sizes so as to optimize surface contact among the chips [26] (Figure 2).

Next the selected definite neck component was permanently seated on the proximal end of the stem. The taper connection between stem and neck components was then tightened with an axial extension bolt using the torque wrench (25 Nm) while the assistant applied counter-torque with the holding device, after which the locking bolt was inserted. After the definitive head was attached, the joint was then reduced.

### Statistical analysis

The analogically recorded values out of the register were digitized and exported to MS Excel 2007 (Microsoft, Redmond, WA, USA) and SPSS 17 (IBM, Armonk, NY, USA). Equality of variance for an unpaired t-test was evaluated in a Levené test. Individual comparisons were analyzed using chi-square cross tabulations and the corresponding Fischer exact test, which determined the likelihood quotient. The Kaplan-Meier survival rates were determined by SPSS 17.

## Results

### Clinical evaluation & complications

There was no significant difference between the study and control groups regarding the mean follow up [4.4 ± 1.7 vs. 4.4 ± 1.8 years, p = 0.93]. The Kaplan-Meier survival probability for the MRP-TITAN stem in both groups together was 93.8% after 8.8 years (95% CI 8.41–9.21). [Study group 95.7% after 8.54 years (95% CI 7.83-9.08); control group 93.1% after 8.7 years (95% CI 8.19-9.20)].

Clinical examination showed significant improvement in the HHS from 35.5 ± 14.3 to 71 ± 9.1 (study group, p < 0.05) and 39.4 ± 15.7 to 70.2 ± 12.2 (control, p < 0.05). The study group showed a significant higher improvement in preoperative vs. postoperative Harris Hip Score (30.8 ± 15.8 vs. 35.6 ± 14.3; p < 0.05). Duration of surgery varied significantly between the two groups (p < 0.05) [232 ± 63.3 min for the study group vs. 186 ± 59.4 min for the control group], while duration of hospitalization, time of mobilization, and time of full weight bearing did not. The data is summarized in Table 2.

**Table 2:** Complication rates

	**Study group**	**Control group**	**p value**
	**(n = 70)**	**(n = 173)**	
**Intraoperative Complications**			
shaft fissure [n] (%)	3 (4.3)	9 (5.2)	0.77
shaft fracture [n] (%)	1 (1.4)	2 (1.2)	0.86
Shaft perforation [n] (%)	2 (2.9)	4 (2.3)	0.80
Avulsion of greater trochanter [n] (%)	1 (1.4)	3 (1.7)	0.87
Sum [n] (%)	7 (10)	18 (10.4)	0.93
**Postoperative Complications**			
Dislocations [n] (%)	5 (7.1)	10 (5.8)	
Closed reduction [n] (%)	2 (2.9)	5 (2.9)	
Open reduction [n] (%)	3 (4.3)	5 (2.9)	
Periprosthetic fracture [n] (%)	1 (1.4)	5 (2.9)	
Aseptic loosening [n] (%)	1 (1.4)	5 (2.9)	
Periprosthetic infections [n] (%)	1 (1.4)	3 (1.7)	
Early infection [n] (%)	0 (0)	0 (0)	
Late infection [n] (%)	1 (1.4)	3 (1.7)	
**Postoperative complications [n] (%)**	**8 (11.4)**	**23 (13.3)**	
**Revision surgery required [n] (%)**	**6 (8.6)**	**19 (11)**	
**Failure rate [n] (%)**	**3 (4.3)**	**8 (4.6)**	

Summary of intraoperative and postoperative complications.

The frequency of intraoperative complications was the same in both groups [10% (n = 7 ) in the study group vs. 10.4% (n = 18) in the control group, (p = 0.93)]. Three shaft fissures (4.3%) occurred in the study group and 9 in the control group (5.2%; p = 0.77). One shaft fracture (1.4%) which occurred intraoperativly in the study group and 2 shaft fractures in the control group (1.2%; p = 0.86) were treated by open reduction and internal fixation. One avulsion fracture of the greater trochanter in the study group and 3 in the control group were reduced and stabilized by tension banding (1.4% vs. 1.7%; p = 0.87).

The rate of postoperative complications did not vary significantly between the study and control groups [n = 8 (11.4%) in the study group vs. n = 23 (13.3%) in the control group, p = 0.69]. Six implants (8.6%) in the study group and 19 implants (11%) in the control group required surgical revision (p = 0.58). The causes for revision in the study group included one nonrecurring periprosthetic late infection after 4.4 years and one periprosthetic fracture occurring as a result of trauma sustained in a fall 5.2 years after implantation. One MRP-TITAN implant was evaluated as showing aseptic loosening with axial migration > 5 mm after 0.5 years on the conventional radiograph. It was replaced with an MRP-TITAN implant with a larger diameter. In the control group, five implants showing aseptic loosening required revision surgery. In 4 of these cases, axial migration of > 5 mm occurred within the first year; one case showed a progressive periprosthetic radiolucency 5.6 years after implantation. In each of these cases the stem was replaced with an MRP-TITAN implant with a larger diameter. Additionally, 5 periprosthetic fractures occurred in trauma sustained in a fall during the follow-up period between the 3rd and 7th years postoperatively. In all of these cases, the stability of implant fixation was uncompromised and the fracture was successfully treated by open reduction and internal fixation.

Periprosthetic late infections occurred in 3 cases. Two of these cases involved reinfection after reimplantation into a Girdlestone hip, occurring 2.7 and 3.1 years after implantation. The other case was a new infection occurring after 3.5 years. A total of 15 postoperative dislocations occurred in the study and control groups [n = 5 in the study group (7.1%); n = 10 in the control group (5.8%), p = 0.69] within a period of 5 weeks to 3 years postoperatively. In 7 cases [n = 2 (2.9%) in the study group; n = 5 (2.9%) control group, p = 0.99] a closed reduction was sufficient, eight patients required open reduction [n = 3 (4.3%) in the study group vs. n = 5 (2.9%) in the control group, p = 0.58]. Within the process of open reduction, six times the length and/or anteversion of the neck component was also altered, without replacing the femoral stem. All complications are summarized in Table 3.

**Table 3:** Clinical outcome data

	**Study group (n = 70)**	**Control group (n = 173)**	**p value**
Time after surgery [years]	4.4 ± 1.7	4.4 ± 1.8	0.93
Duration of operation [min.]	232 ± 63.3	186 ± 59.4	< 0.001
Hospitalization time [d]	24.4 ± 7.3	24.5 ± 6.5	0.94
Mobilization [d]	2.4 ± 1.7	2.1 ± 1	0.16
Full weight bearing [d]	9.8 ± 5,1	10.5 ± 6.8	0.39
HHS postop.	71 ± 9.1	70.2 ± 12.2	0.62
Difference in HHS (preop. vs. postop.)	35.6 ± 14.3	30.8 ± 15.8	<0.05

### Radiographic evaluation

Table 4 summarizes the results of radiologic evaluation. Radiographic evaluation showed full osteointegration of the stem with good contact between implant and bone in 66 patients (94.3%) in the study group and in 156 patients (90.2%) in the control group (p = 0.30). Periprosthetic radiolucencies were demonstrated in 4 cases (5.7%) in the study group and in 17 cases (9.8%) in the control group (p = 0.30). Partial radiolucencies in the proximal zones 1 and 7 according to Gruen occurred significantly more often in the control group without allograft augmentation [n = 15 (8.6%) in the control group vs. n = 1 (1.4%) in the study group vs. p = 0.04].

**Table 4:** Radiographic outcome

	**Study group**	**Control group**	**p value**
	**n = 70**	**n = 173**	
Stable bony union [n] (%)	66 (94.3)	156 (90.2)	0.30
**Periprosthetic radiolucencies**			
No periprosthetic radiolucency [n] (%)	66 (94.3)	156 (90.2)	0.30
Partial radiolucency < 1 mm [n] (%)	2 (2.9)	7 (4)	0.66
Partial radiolucency > 1 mm [n] (%)	2 (2.9)	9 (5.2)	0.43
Progressive radiolucency [n] (%)	0 (0)	1 (0.6)	0.52
**Sum of radiolucencies [n] (%)**	**4 (5.7)**	**17 (9.8)**	**0.30**
**Stem migration**			
No stem migration [n] (%)	67 (95.7)	157 (90.8)	0.19
Stem migration < 2 mm [n] (%)	1 (1.4)	8 (4.6)	0.23
Stem migration 2–5 mm [n] (%)	1 (1.4)	4 (2.3)	0.66
Stem migration > 5 mm [n] (%)	1 (1.4)	4 (2.3)	0.66
**Sum of stem migration [n] (%)**	**3 (4.3)**	**16 (9.2)**	**0.19**
**Proximal stress shielding**			
Proximal stress shielding [n] (%)	4 (5.7)	31 (17.9)	<0.05

Axial implant migration of < 2 mm was seen in one case in the study group as opposed to 8 cases in the control group (p = 0.23), migration of 2–5 mm also in one case in the study group vs. 4 cases (2.3%) in the control group (p = 0.66) and migration of > 5 mm was observed in one case (1.4%) in the study group as opposed to 4 cases (2.3%) in the control group (p = 0.66).

Evaluation of periprosthetic bone remodeling demonstrated a significant reduction in proximal stress shielding in the study group compared with the control group [4 cases (5.7%) vs. 31 cases (17.9%; p < 0.05)]. Secondary stem rotation or varus or valgus tilting of more than 2° was not observed in any of the cases.

**Figure 3:**
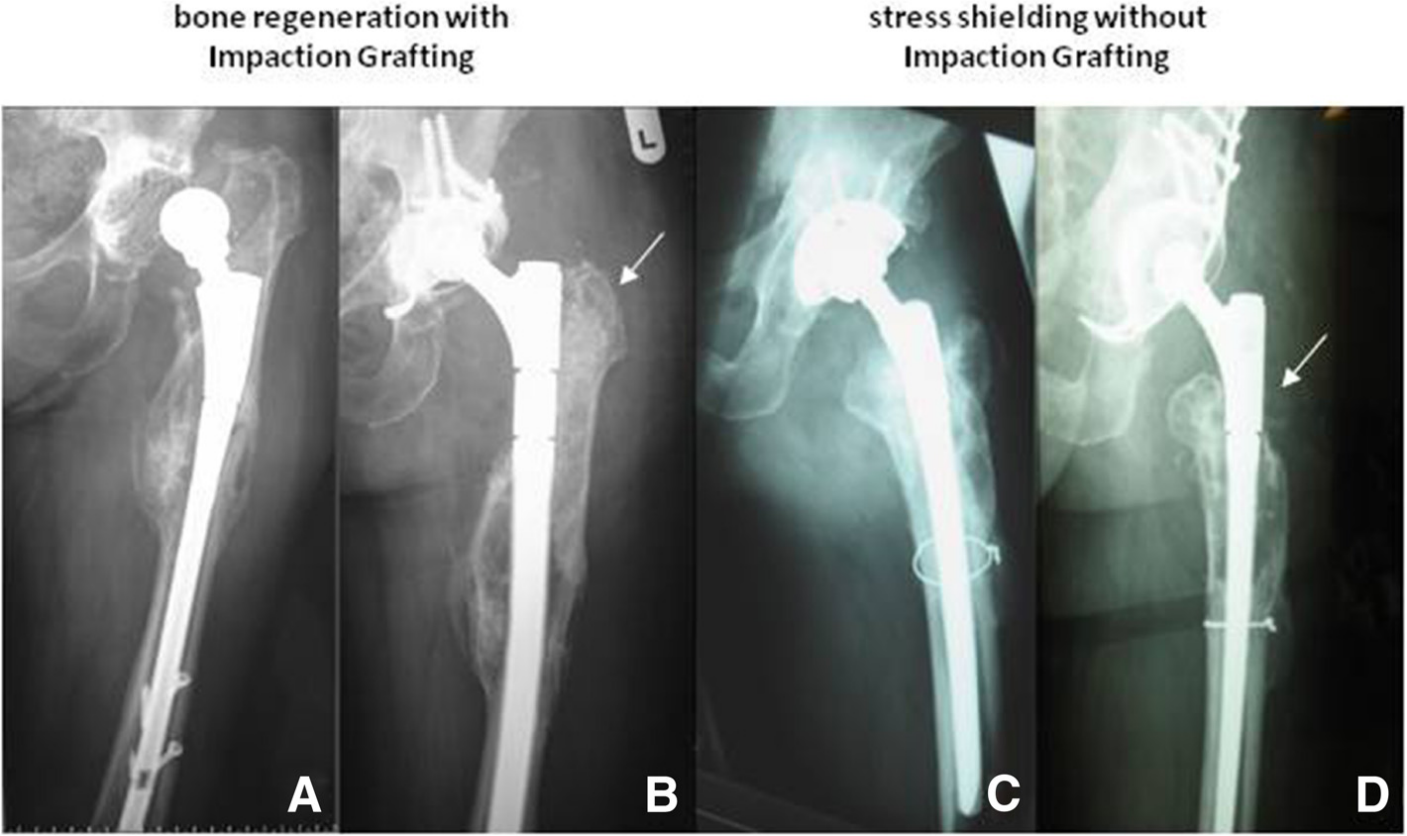
**Radiographic assessment.** Preoperative and two years postoperative follow-up radiographs of revisions with implantation of a MRP-TITAN stem with distal diaphyseal fixation. **3.A**: preoperatively, **3.B**: postoperatively with metaphyseal defect augmentation by impaction bone grafting. 3.B. shows consolidation of the metaphyseal allograft after impaction grafting. **3.C**: preoperatively, **3.D**: postoperatively without impaction bone grafting and stress shielding in the proximal femur.

In the study group receiving allograft augmentation, radiologic evaluation showed complete bony union of the graft (Figure 3) in 66 cases (94.3%). Partial resorption (< 25%) or, respectively, incomplete bony union was demonstrated in only 3 cases (4.3%), whereas resorption > 25% was demonstrated in only one case (1.4%) (Table 5).

**Table 5:** Radiographic graft integration

	**Study group**
Number of hips [n]	70
Graft fixation intact [n] (%)	66 (94.3)
Bony union achieved [n] (%)	66 (94.3)
Osteolytic zone < 2 mm [n] (%)	3 (4.3)
Osteolytic zone > 2 mm [n] (%)	1 (1.4)
Graft resorption ≤ 25 % [n] (%)	3 (4.3)
Graft resorption 25-75 % [n] (%)	1 (1.4)
Graft resorption > 75 % [n] (%)	0 (0)

Evaluation of periprosthetic bone remodeling and regeneration in the study group.

### Clinical and radiographic subgroup evaluation

In a subgroup analysis (Table 6) of patients with large metaphyseal bone defects (types IIC and III as described by Paprosky), the study group showed significantly greater improvement in preoperative vs. postoperative HHS (types I, IIA and IIB: 32.75 ± 13.9 vs. 32.6 ± 15.7, p = 0.89; types IIC and III: 44.6 ± 10.6 vs. 27.3 ± 16.1, p < 0.05).

**Table 6:** Subgroup analysis

**Femoral defects by Paprosky**	**p-values**				
	**I**	**IIA**	**IIB**	**IIC**	**III**
Bony union	1	0.11	0.17	0.07	<0.05
Sum of radiolucencies	1	0.11	0.17	0.07	<0.05
Sum of stem migration	0.07	0.10	0.13	<0.05	0.07
“Stress-Shielding”	0.38	0.70	0.94	<0.05	<0.05
HHS difference [pre- vs. post-OP]	0.34	0.93	0.32	<0.05	<0.05
**Stem - Diameter**	13 / 14 mm	15 / 16 mm	17 / 18 mm	19 / 20 mm	> 21 mm
Bony union	0.16	0.12	0.08	0.08	0.08
Sum of radiolucencies	0.16	0.12	0.08	0.08	0.08
Sum of stem migration	0.16	0.47	0.11	0.08	0.08
“Stress-Shielding”	0.39	0.30	<0.05	<0.05	0.08
HHS differece [pre- vs. post-OP]	0.34	0.93	<0.05	<0.05	<0.05

Detailed evaluation of clinical and radiographic results with consideration given to the femoral defect situation and implant diameter: levels of significance in difference (p-values) between control and study group.

With respect to stem diameter, patients receiving stems with a diameter of 17 mm or larger showed significantly greater improvement in preoperative vs. postoperative HHS when impaction grafting was used (31.5 ± 15.6 vs. 28.6 ± 16.9 to 16 mm, p = 0.36; 41.9 ± 11.7 vs. 30.7 ± 15.1 for 17 mm or larger, p <0.05). These larger stems also showed significantly better radiographic osteointegration when impaction grafting was applied (92.6% vs. 81.5%; p < 0.05), and proximal stress shielding could be reduced significantly in the study group (p ≤ 0.05) (Table 6).

## Discussion

The best strategy to achieve implant stability in revision THA in the presence of extensive metaphyseal defects and with a smooth-walled femoral canal is a distal diaphyseal stem fixation concept [27]. This is especially true for large femoral defects (types IIC and III as described by Paprosky). The deficient proximal bone stock contraindicates a stem fixation concept with metaphyseal stress transfer. Loosening rates of up to 23% have been reported for nonmodular implants with proximal metaphyseal stress transfer after a mean follow-up period of 1.5–13 years [18,19]. The data for this study was collected over an average follow-up period of 4.4 ± 1.8 years (2.1–9.6 years). Based on these results, the cementless MRP-TITAN revision stem with its distal diaphyseal fixation concept has proven effective in femoral revision THA [17,25].

However, diaphyseal fixation can result in proximal stress shielding. This may lead to bone resorption in the non-weight-bearing proximal femur [20]. The present results suggest that reduced stress on the proximal femur with subsequent osteopenia resulting from diaphyseal fixation may be expected primarily in the absence of proximal metaphyseal defect augmentation. Evaluation of periprosthetic bone remodeling showed stress shielding in the form of radiologically verifiable bone resorption in 17.9% of all cases without proximal defect augmentation. Yet this phenomenon was significantly reduced by two thirds when the femoral impaction grafting technique was used.

Andress et al. [28] examined postoperative bone remodeling after implantation of a cementless modular total hip system with initial diaphyseal fixation and without metaphyseal defect augmentation. Quantitative computer tomography (QCT) measured a greater decrease in periprosthetic bone density in the proximal femur after 6 and 12 months than in the distal fixation zone. On average there was a 20.2% decrease compared with preoperative bone density. The study did not demonstrate any correlation between absolute bone density and the relative level of the stress shielding.

In comparison, the follow-up radiographs of the present study suggest that intraoperative allograft augmentation of metaphyseal defects leads to bone regeneration with bone remodeling and stable biologic fixation of the implant. Bony consolidation of the metaphyseal femoral defects with complete bony union of the graft was observed in 94.3% of the cases with supplementary defect augmentation (66 cases). Pronounced bone remodeling in the proximal defect zone in revision arthroplasty with the nonmodular Wagner straight-stem implant has been described in the literature [29-31]. Our examination series failed to find any such remodeling where supplementary metaphyseal grafts were not used.

Our statistical evaluation of the preoperative vs. postoperative clinical function and the postoperative radiologic follow-up suggests that metaphyseal graft augmentation is advisable for large metaphyseal bone defects (Paprosky IIC and III) and stem diameters of 17 mm and larger. The augmentation seems to be able to regenerate the metaphyseal defects, allowing subsequent stress transfer in this region and facilitating integration of larger implants. Clinical function, as expressed in the HHS, is therefore significantly better in the study group when implants with a diameter ≥ 17 mm. Clinical function also showed significantly greater improvement after Impaction Bone Grafting. But with respect to the ranges and the overall outcome and survival, which did not vary significantly, the clinical relevance to our patients of these statistical finding remains questionable.

**Figure 4:**
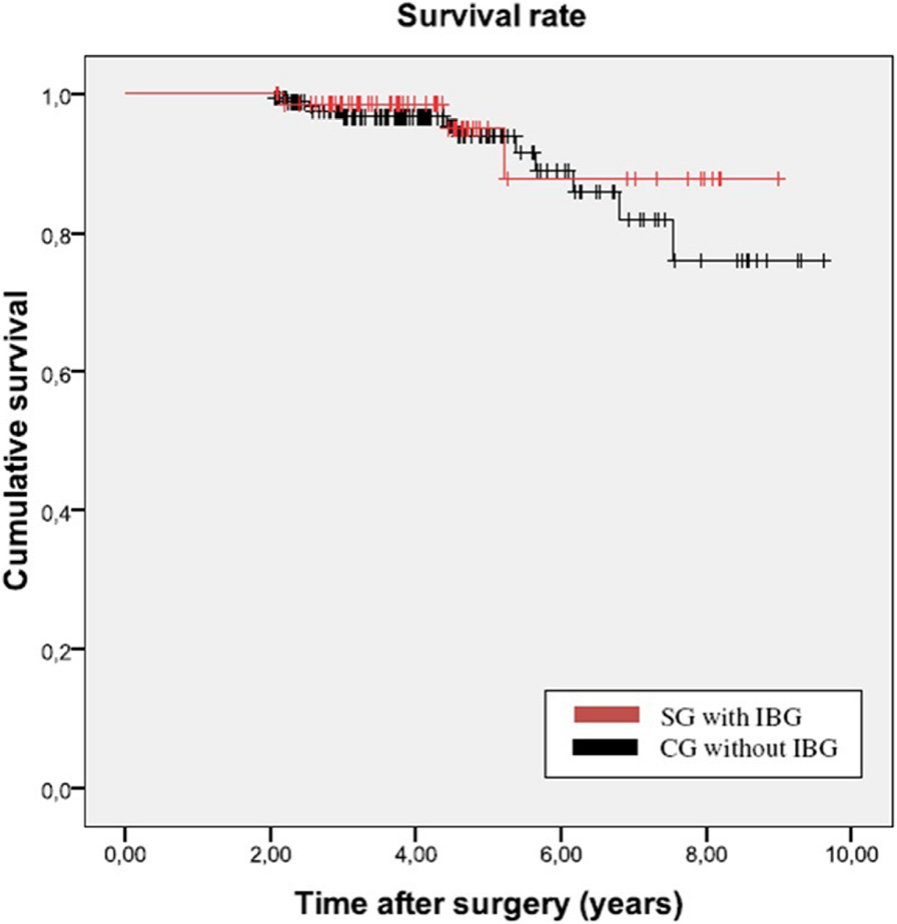
Kaplan-Meier survival rate in the study group and in the control group.

The rate of aseptic loosening was 1.4% (1 case) in the study group and 2.9% (5 cases) in the control group with corresponding Kaplan-Meier survival rates of 95.7% vs. 93.1% after 8.54 and 8.7 years, respectively (Figure 4). Though this does not allow a definitive assessment of the long-term suitability of the MRP-TITAN revision implant, the results appear very encouraging in comparison with reported rates of aseptic loosening of 7-26% for femoral revision THA [32].

Axial implant migration ≥5 mm within one year of surgery without secondary stabilization was observed in a total of 5 cases. Results of the Norwegian Arthroplasty Register show 3 cases of aseptic loosening in a series of 89 femoral revisions within one year of surgery [33]). The cause was presumably an insufficient initial diaphyseal press fit without secondary stabilization. To minimize the risk of axial migration, it is important to use stems in a sufficient diameter in relation to the femoral canal. Short-stemmed implants with larger distal diameters should be preferred to longer, more slender stems. Additionally, care must be taken to achieve proper rotation when using a curved MRP-TITAN stem. A three point fixation instead of the desired diaphyseal press fit fixation has to be avoided. Here the risk of significant subsidence with excessive stress on the bone adjacent to the tip of the stem, leading to a periprosthetic stress fracture [30], can arise.

Implants with axial migration < 5 mm showed radiographic evidence of secondary stabilization within the first year postoperatively without signs of further subsidence during the follow-up period. A comparison with published studies of cementless, nonmodular straight-stem revision implants such as the Wagner implant shows that the axial migration process occurs almost invariably with this implant design. The majority of Wagner implants in the relevant studies showed secondary stabilization after 3–13 months [33,34] with a mean migration rate of 3.2-6.1 mm [33-35]. Boehm and Bischel report on axial migration > 5 mm in 34% of all cases and > 10 mm in 20% in patients with 129 Wagner implants and a mean follow-up of 4.8 years (2 months to 11.1 years) [34].

The fact that the MRP-TITAN implant showed less axial migration compared with the cementless Wagner stem may be caused by the specific geometry of the implant design. The distal support and the press fit in the anterior bow of the femur provide rotational stability in addition to axial support. The crucial factor surgically is to ream the diaphyseal femoral canal to achieve a broad area of contact between implant and cortical bone over a distance of at least 7 cm. This will ensure stable distal fixation. The stellate arrangement of relatively sharp ribs on the MRP-TITAN stem and the tapered design provide sufficient preloading in the often sclerotic diaphyseal bone. This ensures initial axial and rotational stability. This construct, combined with the rough corundum-blasted implant surface facilitates bone ingrowth for secondary biologic fixation.

The number of intraoperative complications did not differ significantly between the study and control group. In particular, there were no significant differences in the numbers of intraoperative shaft fissures/fractures. Compared with rates of up to 21% reported in the literature [36], the rate of shaft fissures/fractures (n = 8 (11.4%) in the study group and n = 24 (13.9%) in the control group) is low. Cerclage wires can help to avoid iatrogenic fractures and fissures while reaming the canal, placing the tapered implant, and impacting the metaphyseal allograft. Especially in fragile and thin cortical bone, the prophylactic placement of cerclage wires in the diaphyseal fixation zone as well as in the metaphyseal augmentation zone should be considered. Within the follow-up the total rate of periprosthetic fractures [n = 1 (1.4%) in the study group vs. n = 5 (2.9%) in the control group] was comparable to those reported in other studies [11,32,37]. In each of these cases the periprosthetic fractures resulted from sufficient trauma. No case was observed in which excessive stresses acting on the bone adjacent to the tip of the implant stem eventually led to periprosthetic stress fractures from three point fixation of the implant.

### Limitations

Our study has potential shortcomings. The decision to use impaction grafting was made intraoperatively based on clinical criteria such as defect size, defect location and containment, rather than using a randomized patient allocation. However, the both groups did not vary significantly according to age, sex, weight, body mass index (BMI), and surgical parameters. Another limitation is that multiple surgeons performed the operations. Even though all of them were experienced attending-surgeons and all of them used the MRP-TITAN stem and the same Impaction Grafting System this might have compromised our results. With a mean follow-up period of 4.4 ± 1.8 years, the present study does not allow a definitive assessment of the long-term results of impaction bone grafting technique. Due to the limitations of a retrospective and descriptive study the level of evidence remains low. A future prospective controlled trial seems essential.

## Conclusion

We present, to the best of our knowledge, the largest cohort on impaction grafting in combination with cementless distal diaphyseal stem fixation published so far. Our hypothesis was that impaction bone grafting improves the survival when using the MRP-TITAN stem. Our analysis provides initial evidence of sufficient bone regeneration with graft augmentation of metaphyseal bone defects. There was a statistical significant reduction in proximal stress shielding and in periprosthetic radiolucencies in zones 1–7 according to Gruen. This corresponded to a significant improvement in clinical function, expressed as the increase in the postoperative HHS.

Our data suggests, that proximal metaphyseal graft augmentation should be considered in revision THA with large metaphyseal bone defects (types IIC and III as described by Paprosky) and with stem diameters of 17 mm and above.

## Competing interests

Peter Brehm GmbH, Weisendorf, Germany, has acted as sponsor in the study presented herein, and finances the article-processing charges for open-access publication. SG and DCW have received research grants from the same institution. The remaining authors declare that they have no competing interests.

## Authors’ contributions

MDW and TMR did the retrospective and descriptive analysis, carried out the statistical analysis of the dataset and drafted the manuscript. MCD, RA, UN, RF and NG contributed by performing patient follow up visits, radiographic analysis, gathering of the data and translation or revision of the manuscript. DCW and SG conceived of the study and its design, conducted the execution and edited the manuscript. All authors read and approved the final manuscript.

## Pre-publication history

The pre-publication history for this paper can be accessed here:

http://www.biomedcentral.com/1471-2474/14/19/prepub
